# Asymptomatic transmission of SARS‐CoV‐2 and implications for mass gatherings

**DOI:** 10.1111/irv.12767

**Published:** 2020-05-30

**Authors:** Justin Wong, Sirajul Adli Jamaludin, Mohammad Fathi Alikhan, Liling Chaw

**Affiliations:** ^1^ Disease Control Division Ministry of Health Bandar Seri Begawan Brunei Darussalam; ^2^ Environmental Health Division Ministry of Health Bandar Seri Begawan Brunei Darussalam; ^3^ PAPRSB Institute of Health Sciences Universiti Brunei Darussalam Gadong Brunei Darussalam

To the Editor

Yu X & Yang R, and others have reported on asymptomatic transmission of SARS‐CoV‐2 at the familial level.^1^, ^2^, ^3^ We also note multiple reports of SARS‐CoV‐2–associated superspreading events (SSEs) arising from mass gatherings; however, the extent of transmission from asymptomatic carriers at these events has not been characterized.^4^, ^5^ Observational studies reporting a high household secondary attack rate and modest R_0_ suggest a transmission dynamic biased toward SSEs.^6^ The presence of asymptomatic carriers could have implications for how relevant authorities handle mass gatherings. We report on two separate settings with presumptive asymptomatic carrier transmission in Brunei Darussalam that can be traced to a single mass gathering in Malaysia.

From February 28 to March 2, 2020, a religious event was held at the Seri Petaling Mosque in Kuala Lumpur, Malaysia, that was later determined to be an international SSE.^5^ This event, now known as the Tablighi Jamaat cluster, was attended by 16 000 people including 75 persons from Brunei, of whom 19 were subsequently confirmed positive for SARS‐CoV‐2 on return from Malaysia. These resulted in a further 52 locally transmitted cases.

We reviewed medical and outbreak investigation records of five cases of coronavirus disease‐19 (COVID‐19) who were part of the Brunei branch of the Tablighi cluster. For diagnosis, nasopharyngeal (NP) samples were collected and tested using real‐time reverse transcriptase polymerase chain reaction (RT‐PCR). On admission, all patients underwent routine blood tests and chest X‐ray. Patients were discharged following resolution of symptoms, and negative RT‐PCR results from two consecutive NP samples collected at ≥24‐hour intervals. This study was approved by the local institutional ethics committee.

Patient A1 (presumed asymptomatic carrier in household setting) is a 30‐year‐old man. On February 27, he traveled to Malaysia to attend the Tablighi event and returned to Brunei on March 3. Following detection of the country's first COVID‐19 case and the subsequent identification of the Tablighi cluster, all participants of that event who returned to Brunei were tested. His NP sample taken on March 10 was positive by RT‐PCR, and he was admitted to the National Isolation Centre. He was asymptomatic on admission and remained so throughout (Figure 1). His blood investigations and chest X‐ray were normal. RT‐PCR was still positive on March 24, and negative on March 26 and 27. All 7 household contacts were tested on March 12, out of which two were found positive. Patient A2 (wife, aged 32) and patient A3 (daughter, aged 10 months) denied any travel or other relevant contact history. Patient A2 reported rhinorrhea which began on March 9; however, this had resolved on presentation. She was asymptomatic throughout her admission. Patient A3 was observed by her mother to have a mild cough starting on March 11, and however was afebrile and had no symptoms on and throughout admission.

**Figure 1:**
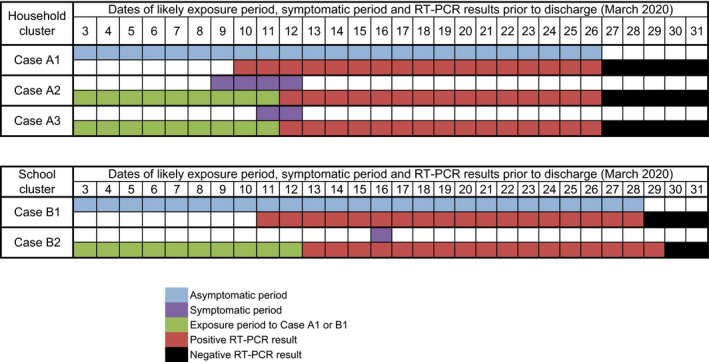
Timeline of presumed transmission from an asymptomatic coronavirus disease‐19 (COVID‐19) case in a household (top) and school (bottom) cluster

Patient B1 (presumed asymptomatic carrier in school setting) is a 13‐year‐old girl. She is an asymptomatic household contact of her father, who was a participant at the same Tablighi event in Malaysia. Her father was confirmed positive for SARS‐CoV‐2 on March 11, and as a contact, her NP sample was collected that same day. She tested positive and was admitted to NIC. She was asymptomatic on presentation and remained so throughout admission (Figure 1). Her blood investigations and chest X‐ray were normal. RT‐PCR was positive on March 27 and negative on March 29 and 30. Contact tracing for case B1 identified 29 school contacts, all of whom were tested. Among them, her class teacher (patient B2, 39‐year‐old female) tested positive on March 12. On admission on March 14, patient B2 reported a mild cough which resolved the next day and has had no further symptoms since.

We report two different settings where the sequence of events is highly suggestive of SARS‐CoV‐2 transmission by asymptomatic carriers, both with clear epidemiological links to a single mass gathering—the Tablighi event. We document how this event has led to at least two further generations due to asymptomatic spread. As far as we are aware, this is the first such report documenting the role of asymptomatic SARS‐CoV‐2 transmission in the propagation of a large superspreading event.

These findings have two key implications. First, cases without symptoms suggest silent chains of transmission. These cases are harder to detect, as they are less likely to present to healthcare facilities, and many testing criteria require the presence of symptoms.^7^, ^8^ Our findings support implementation of testing and longitudinal surveillance of close contacts in the absence of symptoms. This is particularly important if we aim to “find, isolate, test, and treat every case to break the chains of COVID transmission.” ^9^

Second, given the high proportion of asymptomatic individuals (models estimate 30%), our findings strengthen the argument for widespread testing at mass gatherings in areas of known community transmission.^10^ These have the potential to become SSEs, and diagnostic delays can fuel exponential growth.^11^ As countries begin to slowly relax restrictions from community “lockdowns,” widespread testing of participants at mass gatherings with consequent identification of asymptomatic carriers may allow for restrictions to be relaxed in a safe way. Our findings suggest that this large‐scale testing is feasible when deployed reactively after a confirmed COVID‐19 case is identified. Countries may also anticipate that mass gatherings have the potential to become SSE. Assessment of SARS‐CoV‐2 transmission dynamics that better account for asymptomatic transmission at mass gatherings can assist in determining effectiveness of testing participants at these events proactively even before cases are identified, to eliminate transmission at an earlier stage.

## CONFLICT OF INTEREST

None to be declared.

## AUTHOR CONTRIBUTION

**Justin Wong:** Conceptualization (lead); Data curation (lead); Formal analysis (equal); Investigation (equal); Supervision (lead); Visualization (supporting); Writing‐original draft (lead); Writing‐review & editing (equal). **Sirajul Adli Jamaludin:** Data curation (equal); Investigation (equal); Methodology (equal); Supervision (supporting); Writing‐original draft (equal); Writing‐review & editing (equal). **Mohammad Fathi Alikhan:** Data curation (equal); Formal analysis (equal); Investigation (equal); Methodology (equal); Supervision (supporting); Visualization (equal); Writing‐review & editing (equal). **Liling Chaw:** Conceptualization (supporting); Visualization (equal); Writing‐original draft (equal); Writing‐review & editing (equal).

## References

[1] Yu X , Yang R . COVID‐19 transmission through asymptomatic carriers is a challenge to containment. Influenza Other Respir Viruses. 2020. 10.1111/irv.12743 PMC7228388 32246886

[2] Bai Y , Yao L , Wei T , et al. Presumed Asymptomatic carrier transmission of COVID‐19. JAMA. 2020;323(14):1406. 32083643 10.1001/jama.2020.2565 PMC7042844

[3] Li C , Ji F , Wang L , Wang L . Asymptomatic and human‐to‐human transmission of SARS‐CoV‐2 in a 2‐family cluster, Xuzhou, China. Emerg Infect Dis. 2020;26:7. 10.3201/eid2607.200718 PMC7323514 32228809

[4] Kim HJ , Hwang HS , Choi YH , et al. The delay in COVID‐19 confirmation among cases from a religious group in South Korea. J Prevent Med Public Health. 2020. 10.3961/jpmph.20.088 PMC7280806 32498138

[5] Mat NFC , Edinur HA , Razab MKAA , et al. A single mass gathering resulted in massive transmission of COVID‐19 infections in Malaysia with further international spread. J Travel Med. 2020;27(3). 10.1093/jtm/taaa059 PMC7188142 32307549

[6] Liu Y , Ego RM , Kucharski AJ . Secondary attack rate and superspreading events for SARS‐CoV‐2. The Lancet. 2020;395:e47. 10.1016/S0140-6736(20)30462-1 PMC7158947 32113505

[7] World Health Organization . Global surveillance for COVID‐19 caused by human infection with COVID‐19 virus. Interim Guidance. March 20, 2020.

[8] Public Health England . COVID‐19: investigation and initial clinical management of possible cases. April 6, 2020 update. https://www.gov.uk/government/publications/wuhan-novel-coronavirus-initial-investigation-of-possible-cases/investigation-and-initial-clinical-management-of-possible-cases-of-wuhan-novel-coronavirus-wn-cov-infection (accessed April 22, 2020).

[9] WHO Director‐General's opening remarks at the media briefing on COVID‐19 ‐ 13 March 2020. [Internet], 2020. https://www.who.int/dg/speeches/detail/who-director-general-s-opening-remarks-at-the-mission-briefing-on-covid-19---13-march-2020 (accessed April 22, 2020)

[10] Nishiura H , Tetsuro Kobayashi T , Ayako Suzuki A , et al. Estimation of the asymptomatic ratio of novel coronavirus infections (COVID‐19). Int J Infect Dis. 2020;94:154‐155. 32179137 10.1016/j.ijid.2020.03.020 PMC7270890

[11] Frieden TR , Lee CT . Identifying and interrupting superspreading events—implications for control of severe acute respiratory syndrome coronavirus 2. Emerg Infect Dis. 2020;26:1059‐1066. 32187007 10.3201/eid2606.200495 PMC7258476

